# Spectral sensitivity near exceptional points as a resource for hardware encryption

**DOI:** 10.1038/s41467-023-36508-x

**Published:** 2023-02-28

**Authors:** Minye Yang, Liang Zhu, Qi Zhong, Ramy El-Ganainy, Pai-Yen Chen

**Affiliations:** 1 Department of Electrical and Computer Engineering, University of Illinois Chicago, Chicago, IL 60607 USA; 2 Department of Physics, Michigan Technological University, Houghton, MI 49931 USA; 3 Henes Center for Quantum Phenomena, Michigan Technological University, Houghton, MI 49931 USA

**Keywords:** Electrical and electronic engineering, Electronics, photonics and device physics

## Abstract

The spectral sensitivity near exceptional points (EPs) has been recently explored as an avenue for building sensors with enhanced sensitivity. However, to date, it is not clear whether this class of sensors does indeed outperform traditional sensors in terms of signal-to-noise ratio. In this work, we investigate the spectral sensitivity associated with EPs under a different lens and propose to utilize it as a resource for hardware security. In particular, we introduce a physically unclonable function (PUF) based on analogue electronic circuits that benefit from the drastic eigenvalues bifurcation near a divergent exceptional point to enhance the stochastic entropy caused by inherent parameter fluctuations in electronic components. This in turn results in a perfect entropy source for the generation of encryption keys encoded in analog electrical signals. This lightweight and robust analog-PUF structure may lead to a variety of unforeseen securities and anti-counterfeiting applications in radio-frequency fingerprinting and wireless communications.

## Abstract (web summary)

In this work the authors demonstrate that the spectral sensitivity near exceptional points can be harnessed in electronic circuits to implement physically unclonable functions for security and communication applications.

## Introduction

Over the past decade, the physics of exceptional points (EPs) have attracted considerable attention due to their exotic effects and potential applications, mainly in optics and photonics^1–3^, and electronics^4–6^. An EP is formed when two or more eigenstates (eigenvalues and corresponding eigenvectors) of a non-Hermitian Hamiltonian coalesce and become identical^7^. The onset of this peculiar degeneracy signals the collapse of the eigenspace dimensionality which in turn enhances the system’s sensitivity to perturbations. This observation has inspired the recent proposal of building sensing devices operating at EPs^8^. Subsequent experimental studies have indeed confirmed that EP-based sensors exhibit enhanced responsivity^9–11^. However, careful theoretical analysis^12–14^ and experimental results^15^ have raised doubts about the performance of these devices in terms of sensitivity, defined by the signal-to-noise ratio. In particular, it was argued that while indeed the presence of EP leads to enhancement in the responsivity, at the same time it also amplifies the noise by exactly the same factor. On the other hand, very recent experimental results on EP-based mechanical accelerators suggest that there exists a regime where the signal enhancement outweighs that of the noise, thus showing the merit of using EPs for sensing applications^16^. Beyond this active debate, another problem related to the EP-based sensors studied so far, is that they rely on an implementation of isolated exceptional points. This poses a practical challenge because those systems then become very susceptible to fabrication error and noisy environments which degrade their performance. For instance, in the two experiments in refs. ^9,10^, active tuning parameters were employed after the fabrication in order to fine-tune the system to the EP. In this work, we show that this adverse effect (i.e., extreme sensitivity to perturbation near EPs), which is considered as a foe for sensing applications, can in fact present a solution to another urgent problem, namely that of security and authentication. In particular, we demonstrate that naturally occurring fabrication errors can be used to build EP-based electronic circuits, implementing physically unclonable functions (PUFs) with excellent statistical characteristics in terms of the entropy of the generated keys and the uniqueness between different keys.

Traditional security schemes rely on encrypted keys stored inside memory chips. These, however, can in principle be attacked which poses a serious security challenge in almost every aspect of modern life, including safety^17^, authentication of goods, foods, and drugs^18,19^, radio-frequency identification (RFID) authorizations^20,21^, and encrypted communications^22,23^. In this context, internet-of-things (IoTs) systems in which various data such as location, finances, and health are constantly collected by sensors and different electronic and tracking devices through near-field communication (NFC) interface (built in, for example, mobile devices), are particularly vulnerable to such a problem^24–26^. To make things worse, the recent progress in artificial intelligence has made it possible to decrypt some current software-based cryptographic algorithms by using machine/deep learning-assisted attacks^27,28^. Against this backdrop, the idea to use PUFs has emerged among the most promising and cost-effective hardware security primitives for key generations and authentications in cyberspace^29–32^. In general, PUFs exploit unique physical variations that occur naturally during the device manufacturing process, and the encrypted key is generated by mapping a given input (i.e., “challenge”) to an output (i.e., “response”), forming a challenge-response pair (CRP) (e.g., electrical signals in time/frequency domain, mechanical or optical signals). Typically, PUFs can be categorized into two major classes, the strong PUFs capable of generating massive amounts of CRPs, and the weak PUFs possessing only a limited number of CRPs. More explicitly, the CRP number of strong PUFs would grow exponentially with a linear increase of device size, while that of weak PUFs increases only linearly^33^. To date, the majority of PUFs are primarily based on digital electronics, i.e., complementary metal-oxide semiconductor (CMOS) integrated circuit (IC) technologies, including arbiter PUFs^34,35^, static random-access memory (SRAM) PUFs^34–36^, memristor PUFs^37–39^, and ring oscillator PUFs^40–43^. Although CMOS digital products can have good robustness through micro-/nano-manufacturing with high precision, their applications in PUFs, on the flip side, are usually affected by relatively low entropy and power consumption. As a result, CMOS-based PUFs are still potentially vulnerable to machine learning attacks based on predictive regression models and generative adversarial neural networks^44,45^. Other emerging PUFs with improved randomness, such as quantum electronic PUFs^46^, optical and photonic PUFs^47,48^, and those based on features of randomly distributed nanostructures^49^, are still subject to the implementation cost and system complexity.

Here, we demonstrate that typical variations in the values of standard electronic components (resistors, capacitors, and inductors) can be “amplified” when used to build an electric circuit operating at a special type of EPs that also involve pole singularity, which are known as divergent EPs or DEPs. This peculiar non-Hermitian singularity was recently proposed and experimentally demonstrated in ref. ^50^. Our work paves the way for building a new generation of hardware-based encryption architectures that outperforms previous PUFs schemes.

## Results

### Extreme sensitivity of DEP-based circuits

When a resonant Hermitian system (or even non-Hermitian systems operating away from EPs) is subject to perturbation of order *ϵ*, its eigenvalues (resonant frequencies for example in the case of resonators) undergo a shift that scales linearly with perturbation parameter. In contrast, the shift of the degenerate eigenvalue associated with an EP of order $\sqrt{N}$ due to the same perturbation can scale as ${{\Delta }}\omega \sim \root N \of {\epsilon }$^51^. In addition, if the spectrum exhibits a pole singularity such that the eigenvalue shift takes the form ${{\Delta }}\omega \sim \frac{\root N \, \of {\epsilon }}{\sqrt{1-{x}^{2}}}$ for some additional degree of freedom *x*, then it is reasonable to expect further enhancement of the sensitivity in the regime *x* ~ 1. As we have mentioned earlier, this enhanced sensitivity of eigenvalues splitting at/near EPs inspired numerous investigations into their utility for sensing applications. However, at the same time, this same sensitivity to perturbations raised several questions as to the performance of these systems when noise is accounted for. To date, the sensitivity of EP-based sensors, as quantified by their signal-to-noise ratio, is a topic of active debate without final consensus.

**Fig. 1:**
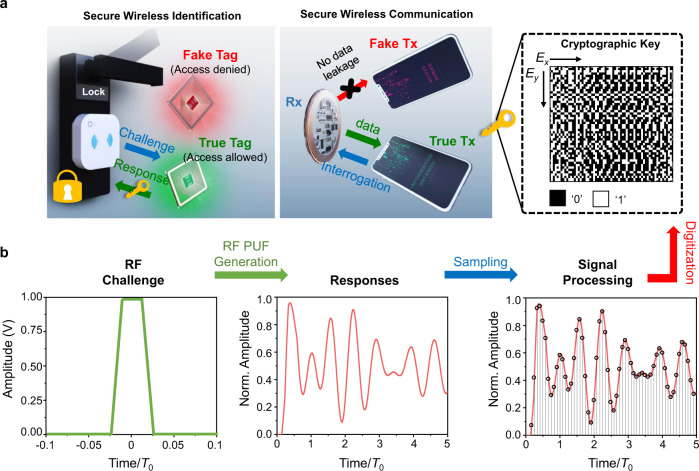
Physically unclonable function (PUF) based cryptographic keys generated by the PT-symmetric electronic system. **a** Illustration of the PUF-enabled secure radio-frequency (RF) authentication and communication. **b** Generation of the challenge-response pair (CRP) and the cryptographic key in the proposed PUF system; here, ${f}_{0}=\frac{1}{2\pi \sqrt{{{{{LC}}}}}}$ is the natural frequency of the “LC” oscillator and *T*_0_ = 1/*f*_0_. Our experiments utilize the pulse excitation shown in the left panel of **b**, and the response, represented by the transient voltage signal measured across the reader’s capacitor, and its discretized form are shown in the middle and right panels of **b**, respectively. After proper sampling and processing, the analog response is converted to a digital key composed of a bitstring.

Here we consider the extreme eigenvalue sensitivity of systems with EP from a different point of view and demonstrate their utility for security applications. In particular, we show that DEP-based circuits are excellent candidates for building robust radio-frequency (RF) PUF structures, which can be generalized to realize secure wireless authentication (e.g., RFID and wireless access control) and NFC systems. Figure 1a depicts the generic architecture of PUF-enabled RF wireless identification and communication systems. The security keys in these systems are sourced from the unavoidable, irreproducible fabrication errors in the values of the electronic components (resistors, capacitors, and/or inductors) that are used to build the receiver circuit, denoted by Rx. These fabrication errors equip each individual circuit with a unique fingerprint that serves as a PUF-based cryptographic key, which can be probed as follows. When the reader and tag are paired for PUF encryption as a secure wireless identification system, the reader (transmitter circuit or Tx) launches an RF pulse, known as a “challenge” to stimulate the Rx receiver. The temporal response of the latter strongly depends on its eigenmodes of the combined system, which in turn are functions of the values of its electronic components. Hence, different tags will exhibit unique temporal responses as given by the instantaneous voltages measured across the reader’s capacitor, called for short as the “response”. Next, the temporal response is digitized to generate a 256-bit identifier (ID) for a given challenge. This process is illustrated in Fig. 1b and details can be found in Supplementary Note 1. Once this digitized ID passes the validation by a specific IoT database via the Tx, the access request of the Rx (tag) will be authorized. On the other hand, when this reader–tag scheme is used for secure wireless communication, the RF signals transmitted by Tx will introduce a unique voltage response drop across the Rx’s capacitor in the time domain. Only when such a temporal response is digitized and verified by Rx with pre-defined verification, the encrypted data and/or information stored in the Rx’s memory will be allowed to be transmitted back to Tx. The secure wireless communication is therefore achieved, effectively avoiding the disclosure of privacy.

**Fig. 2:**
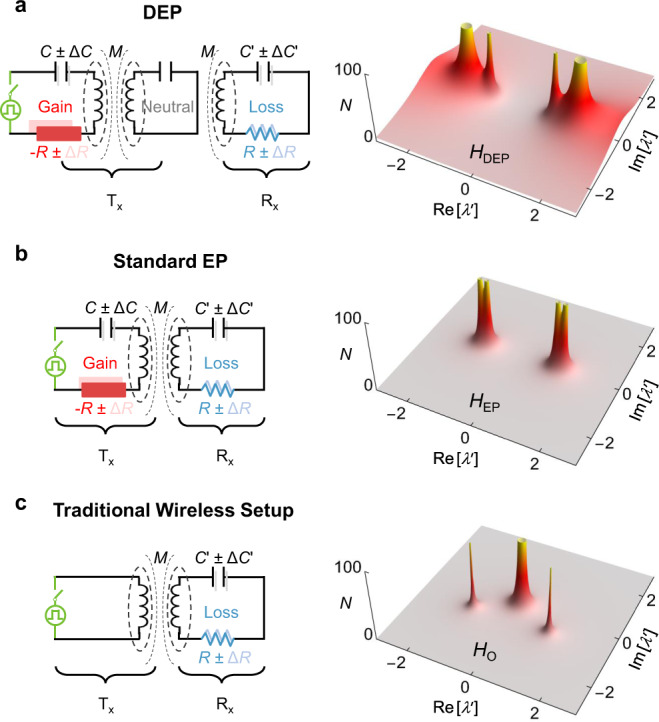
Pseudospectra of non-Hermitian circuits. Transmitter–Receiver (Tx–Rx) architecture that implements a DEP (**a**), a standard EP (**b**), and a traditional near-field telemetry setup (coil antenna) without any EPs (**c**). The corresponding pseudospectra are plotted on the right panel.

In this work, we focus on a Tx–Rx architecture that implements DEPs as shown in left panel of Fig. 2a, which consists of an active transmitter (“−RLC” oscillator), one or multiple neutral intermediator (“LC” oscillator), and a passive receiver (“RLC” oscillator)^50,52–54^. In addition, to evaluate the performances of these DEP-based PUFs, we will compare their performance in terms of security metrics with other circuits that realize EPs as well as non-EP systems, both shown in the left panels of Fig. 2b, c. We start by comparing the eigenvalue sensitivity associated with the three different circuit architectures shown in the left panels of Fig. 2, which implements the above cases. By using Kirchhoff laws, it is straightforward to derive the Hamiltonians that describe these circuits, which we will denote by *H*_DEP_, *H*_EP_, and *H*_O_, respectively. The exact form of *H*_DEP_ is presented in Supplementary Note 2. To compare the sensitivity of these systems against variation of the Hamiltonian parameters, we employ the concept of pseudospectrum^55^. The *ϵ*-pseudospectrum of a matrix *A*, denoted as *σ*_*ϵ*_(*A*), is typically defined as ${\sigma }_{\epsilon }(A)=\{{\lambda }^{{\prime} }\in {\mathbb{C}}:{\lambda }^{{\prime} }\in \sigma (A+E):||E||\le \epsilon \}$. Here *σ*(*A*) denotes the eigenvalue spectrum of *A* and ∣∣...∣∣ is a matrix norm. Basically, it is a measure of how the eigenvalues of the original system vary in response to small perturbations. However, the above definition, which was used in connection with non-Hermitian photonics in ref. ^56^ is difficult to apply. Another equivalent, yet more practical definition, which has been also proven useful in studying PT-symmetric optical systems^57^, is:

1 $${\sigma }_{\epsilon }(A)=\left\{{\lambda }^{{\prime} }\in {\mathbb{C}}:||{(A-{\lambda }^{{\prime} }I)}^{-1}||\ge \frac{1}{\epsilon }\right\},$$

where *I* is the unitary matrix. Here we adopt this latter definition. The right panels of Fig. 2 depict the function $N({\lambda }^{{\prime} })=||H-{\lambda }^{{\prime} }I|{|}^{-1}$ in the complex ${\lambda }^{{\prime} }$ plane for *H*_DEP_, *H*_EP_, and *H*_O_. From these plots, we observe large values of ${N}_{{{{{{{{\rm{DEP}}}}}}}}}({\lambda }^{{\prime} })$ are spread over a larger area in the complex ${\lambda }^{{\prime} }$ plane than that of ${N}_{{{{{{{{\rm{EP}}}}}}}}}({\lambda }^{{\prime} })$, with both exceeding the spread associated with ${N}_{{{{{{{{\rm{O}}}}}}}}}({\lambda }^{{\prime} })$. These plots thus confirm the extreme eigenvalue sensitivity associated with the DEP as compared with conventional EPs, which in turn exceeds that of systems with no EP at all.

The extreme sensitivity of the DEP circuit can be better understood by closely inspecting its eigenfrequencies, which we express here in the unit of the natural frequency ${\omega }_{0}=1/\sqrt{{{{{LC}}}}}$, as discussed in detail in Supplementary Note 2:

2 $${\omega }_{1}=1,\,{\omega }_{\pm }=\,\sqrt{\frac{1-2{\gamma }^{2}\pm \sqrt{1-4{\gamma }^{2}+8{\gamma }^{4}{\kappa }^{2}}}{2{\gamma }^{2}(2{\kappa }^{2}-1)}},$$

where the dimensionless non-Hermitian (expressing gain or loss) parameter and the normalized coupling factor are given by $\gamma={R}^{-1}\sqrt{L/C}$ and *κ* = *M*/*L*, respectively. In these formulas, *L* and *M* are self and mutual inductances of the two coil antennas. From Eq. (2), it is straightforward to check that two bifurcating real eigenfrequencies, *ω*_±_ become degenerate at ${\gamma }_{EP}=1/\sqrt{2}$. Further analysis in ref. ^50^ confirms that the corresponding eigenstates become also identical, i.e., this point is indeed an EP. In addition, the point $\kappa=1/\sqrt{2}$ represents a pole singularity, at which the eigenfrequencies diverge. In reality, however, the nonlinearity of the circuit will eventually regulate this divergent behavior. However, here we consider the system in an intermediate regime where the singularity enhances the eigenfrequency splitting but without causing any divergence, and hence the system can be well studied within the context of linear circuit theory. This DEP divides the system into exact and broken PT symmetry phases^1,53^. Let us now consider a system designed to operate exactly at the DEP. Due to the strong bifurcation around this point, any small deviations in the values of the circuit’s components can lead to a substantial drift in the eigenfrequencies and consequently the response to external excitations. This is exactly the basis for our proposal that utilizes DEP systems for producing a high-performance PUF leveraging the process variation naturally occurring in electronic components.

**Fig. 3:**
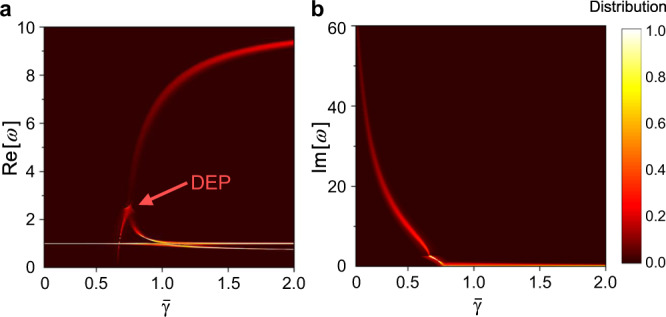
Complex eigenfrequencies of the third-order PT-symmetric circuit. Distributions of real (**a**) and imaginary (**b**) parts of eigenfrequencies for the third-order PT telemetry system (a DEP in a third-order PT telemetry system); here, the same reader (“−RLC” oscillator) is used to interrogate 500 different tags (“RLC” oscillator) whose resistance and capacitance have a Gaussian distribution with a standard deviation *σ* = 0.04. Here, $\bar{\gamma}$ is the mean value of the Gaussian distribution of ${\gamma}$.

To elucidate the effect of random physical variations on the system’s eigenspectrum, we proceed by considering a realistic scenario. It is well known that fabrication errors can lead to a typical variation in the values of electronic components in the range of ±0.001 ~ 0.05^58,59^; such values are close to percentage errors found in realistic electronic components (±0.1 ~ 5%). In line with this, here we consider an ensemble of DEP-circuits (see Fig. 2a) where resistors and capacitors at the receivers end (defined by the variables *R* and *C*) follow Gaussian (normal) distribution given by $P(x)=\frac{1}{\sqrt{2\pi }\sigma }{e}^{-{(x-\mu )}^{2}/2{\sigma }^{2}}$, where *μ* is the mean resistance or capacitance value and *σ* is the standard deviation induced by fabrication errors, which we take it to be *σ* = 0.04 throughout our study for PUF evaluations (applicable for most chip resistors and chip capacitors^60^). Figure 3a, b plots distributions of real and imaginary parts of eigenfrequencies as a function of the *γ* (with the abovementioned uncertainty in electronic components −*R*, *R*, *L*, and *C*) under coupling coefficient *κ* = 0.7. It can be observed from Fig. 3a that real parts of eigenfrequencies are randomly distributed around the DEP, showing high uncertainty and a dark region that infers a low probability of detection. On the contrary, when the system is operated away from its DEP, real parts of eigenfrequencies have a narrow distribution centered at the mean value, as indicated by brighter colors (i.e., high probabilities) in Fig. 3a. In addition, imaginary parts of eigenfrequencies shown in Fig. 3b has a high probability of being zero in the exact PT phase. This statistical analysis clearly shows that even a typical 4% standard deviation in resistance and capacitance values results in a highly random eigenspectrum in the vicinity of DEP, thereby providing an ideal entropy source for PUF and true random number generator applications.

### Experimental validation of DEP-based PUFs

**Fig. 4:**
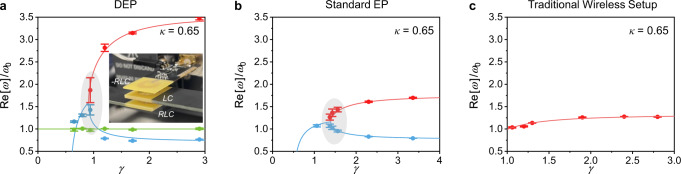
Experimental demonstration of high uncertainty near the DEP. Theoretical (solid lines) and experimentally measured (points) eigenspectra for DEPs (**a**), standard EPs (**b**), and traditional telemetry setups (**c**), whose equivalent circuits correspond to Fig. 2a–c, respectively; the error bars encompass the lowest and highest values measured from 4 PUF instances and the inset of **a** shows the experimental setup of the DEP-based RF PUF prototype.

Having established the extreme spectral sensitivity of DEP-based electronic systems operating at or near their EPs, we now assess their performance when used as RF PUF for contactless identification purposes as shown in Fig. 1a. In the practical implementation, the reader launches the pulse signal(s) of a specific shape as the input challenge(s). Together with the receiver, the whole circuit forms the third-order PT-symmetric electronic system. When the system is turned on, the voltage across the reader’s capacitor can be exploited to extract the security key to enable wireless access control. Finally, the detected transient voltage response over a period is compared with the pre-stored PUF key dataset to determine whether the user access is allowed or denied. To validate this idea, we manufactured 16 tags (“RLC” oscillators) and associated readers for different telemetry setups sketched in Fig. 2. In this work, electronic circuits were realized using the printed circuit board (PCB) techniques and the standard photolithography. The experimental setup is depicted in the inset of Fig. 4a, where *κ* = 0.65 = 0.92*κ*_DEP_; see “Methods” for details on reader and tag design and wireless measurement setup. Figure 4a–c shows the theoretical and measured eigenspectra for the DEP-based, EP-based, and traditional telemetric systems, which correspond to the schematics in Fig. 2a–c. We find that the traditional telemetry setup without any pole-singularity (Fig. 4c) has the smallest error bars, which, for PUF applications, could result in poor randomness and uniqueness of output signals. On the contrary, a long error bar that hints at a huge discrepancy among devices is observed around the DEP (Fig. 4a). The eigenfrequency variation around the EP in somewhere between the two extremes (Fig. 4b). Such results are in good agreement with theoretical predictions in Fig. 3a, suggesting that the DEP-based PUF may have superior PUF randomness and uniqueness.

Next, we consider two standard PUF metrics, namely entropy, and uniqueness associated with the Hamming Distance (HD)^61^. The first indicator quantifies the randomness of the bits generated for a single challenge and different devices, while the latter quantifies how each device is distinct from another. One of the minimal requirements of PUFs is the randomness of their keys. Ideally, the bitmap extracted from the transient voltage responses should have an unbiased distribution of “0” and “1” states. A highly random two-dimensional bitmap (such as that shown in Fig. 1b) distribution is characterized by a high entropy pair (*E*_*x*_, *E*_*y*_) defined by^62^:

3 $${E}_{x} 	=-[{p}_{x}{\log }_{2}{p}_{x}+(1-{p}_{x}){\log }_{2}(1-{p}_{x})],\\ {E}_{y} 	=-[{p}_{y}{\log }_{2}{p}_{y}+(1-{p}_{y}){\log }_{2}(1-{p}_{y})],$$

where *p*_*x*_, *p*_*y*_ are the probabilities of obtaining the digit “1”, along the *x*- and *y*-axis, respectively. For an ideal random source, the distributions of “1” (*p*_*x*_, *p*_*y*_) and “0” (1 − *p*_*x*_, 1 − *p*_*y*_) in a bitstring are both expected to be 50%, resulting in the maximum entropy *E*_*x*,*y*_ = 1, i.e., both are unity. The bitmap generated using the DEP is shown in the inset of Fig. 1a (white means 1 and black means 0). Figure 5a–c reports the entropy functions *E*_*x*_ and *E*_*y*_ for the DEP-based, EP-based, and traditional telemetry systems, respectively. We find that near-ideal entropies can be obtained using the DEP-based PUF system (*E*_*x*_ = 0.93 ± 0.09, *E*_*y*_ = 0.99 ± 0.01). We also compare the performance of DEP-based PUF systems with those that implement a standard EP (i.e., without the pole singularity) as well as those that do not rely on EPs at all (all shown in Fig. 2) from the experimental results. It is clearly seen from Fig. 5a–c that the DEP-based RF PUF device can notably outperform the non-EP device (*E*_*x*_ = 0.43 ± 0.05, *E*_*y*_ = 0.23 ± 0.310) and the EP-based PUF system (*E*_*x*_ = 0.94 ± 0.07, *E*_*y*_ = 0.96 ± 0.08). It is worth mentioning here that the entropy contents are very much straightforward but do not have sufficient parameters to acknowledge the randomness. Therefore, we also employed the National Institute of Standards and Technology (NIST) randomness tests suite^63^ to characterize the randomness of the three PUFs, which has more rigorous standards to verify the randomness of the PUF, with results reported in the Supplementary Note 4. The NIST randomness test results show that only the DEP-based PUF can pass all randomness tests.

In addition to the randomness evaluation, uniqueness indicated by the inter-device HDs^64^ (defined as the counts of different bits between two CRPs under the same challenge) is another important figure of metric. It is worthwhile noting that the device uniqueness is also regarded as the degree of correlation between the one-dimensional digitized keys of two different PUFs. The one-dimensional keys from any two different PUF units, if possible, should be uncorrelated with a normalized inter-HD equaling to 0.5. A long or short normalized inter-HD between two CRPs would deteriorate the quality of encryption, such that one could decipher an unknown CRP from another known CRP. Ideally, the mean inter-HD should be 0.5, which occurs when on average half of the bit length, whereas the mean intra-HD should be close to 0, and its expression is given by:

4 $$\overline{{{{{{{{{\rm{HD}}}}}}}}}_{{{{{{{{\rm{inter}}}}}}}}}}=\frac{2}{N(N-1)}\mathop{\sum }\limits_{i=1}^{N-1}\mathop{\sum }\limits_{j=i+1}^{N}\frac{\mathop{\sum }\nolimits_{l=1}^{L}\left({K}_{i,l}\bigoplus {K}_{j,l}\right)}{L},$$

where *N* represents the number of PUF devices (*N* = 16), *L* is the length of bitstrings digitized from the RF signal (*L* = 256), *K*_*i*_ is the *i*th PUF key, ⨁ denotes the XOR logic operation. Figure 5d–f plots the inter-device HDs for RF PUFs generated by three different systems in Fig. 4; here, the original HD is normalized by the length of the bitstring. The inter-HD of the DEP-based RF PUF can be excellently fitted by a Gaussian distribution centered at *μ* = 0.5006 having the standard deviation *σ* = 0.0277. This indicates that the DEP-based RF PUF devices do indeed exhibit unique responses. On the contrary, from Fig. 5f, we observe that the traditional telemetry setup (dark purple histogram) acts as a low-entropy source with a biased distribution of “1” and “0”, causing the mean inter-HD to downshift to 0 and thus worst-case uniqueness. The EP-enabled bifurcation effect in the standard PT electronic system though can increase the entropy, however, its performance still lags far behind that of the third-order PT electronic system operating nearby the DEP. The distribution of HDs of EP-based PUF (Fig. 5e) is wider than that of DEP PUF, resulting in a larger standard deviation and thus a smaller encoding capacity in the encryption process. Therefore, the EP-based PUF is not as good as the DEP-based one in terms of randomness and uniqueness. The upshot of this comparison (Fig. 5d–f) is that using DEP-based electronic circuits to implement PUF devices can significantly boost the system’s entropy and uniqueness beyond their values in standard devices.

**Fig. 5:**
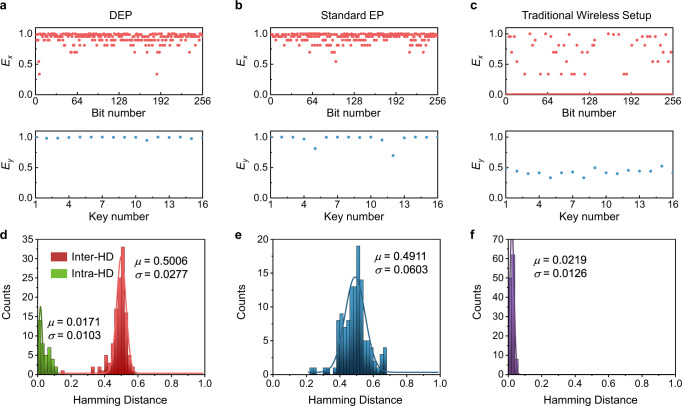
Statistical evaluation of PUFs. Entropy (*E*_*x*_, *E*_*y*_) of the 256-bits PUF response from 16 manufactured PUF instances for the DEP-based (**a**), EP-based (**b**), and traditional RF PUF system (**c**) used for the identification application. **d**–**f** are similar to **a**–**c**, but for the inter-Hamming Distances (inter-HDs) histograms obtained from the same PUF instances. The green histogram in **d** depict the intra-Hamming Distances (intra-HDs) obtained from 4 DEP-based PUF instances that were measured between −20 and 80 °C in an interval of 10 °C.

Another important metric characterizing the performance of PUF devices is their reliability, which is defined as the ability of generating identical keys after the same repeated challenges. In other words, the response associated with the same challenge does not change over time, even though the environmental conditions are changed (e.g., temperatures in electronic components). A reliable RF PUF system should have sufficient tolerance against temperature variation. In general, chip-based RF elements can have constant impedance and low noise over a wide temperature range (−40–80 °C)^65,66^, and, unlike PUFs based on nanomaterial and nanophotonic devices^29,48^, protective packages for electronic components can prevent PUF devices from oxidization and contamination from physical, chemical, and biological sources. Thus, RF PUFs based on the printed circuit board or on-chip integrated circuit technologies can be quite robust and reliable. To verify the key reproducibility, we compare intra-HDs obtained from 11 temperature conditions from −20–80 °C, with its expression given by:

5 $$\overline{{{{{{{{{\rm{HD}}}}}}}}}_{{{{{{{{\rm{intra}}}}}}}}}}=\frac{1}{M}\mathop{\sum }\limits_{q=1}^{M}\frac{\mathop{\sum }\nolimits_{l=1}^{L}({K}_{l}\bigoplus {K}_{q,l})}{L},$$

where *M* is the number of repeated measurements under different environmental conditions. The results reported in Fig. 5d by green histograms show that the distribution of intra-HDs, evaluated at different temperatures, is centered at *μ* = 0.0171 with the standard deviation *σ* = 0.0103, which is close to the ideal case with zero mean value, showing a robust temperature stability it may have. We find that for the DEP-based RF PUF, the mean intra-HD calculated based on Eq. (5) is only 0.04. Such a low mean intra-HD validates good robustness against temperature variations. We note that the reliability can be further improved by using electronic components with lower temperature coefficients, such as those based on specific alloys with buffered substrate. In our experiment, passive and active electronic components used on readers and tags are commercially available and inexpensive. Also, the reader and tag designs are compatible to the NFC framework and can be massively produced at ultralow cost using standard inkjet printing or lithography-based PCB manufacturing methods.

### Large-scale simulations of DEP-based PUFs

**Fig. 6:**
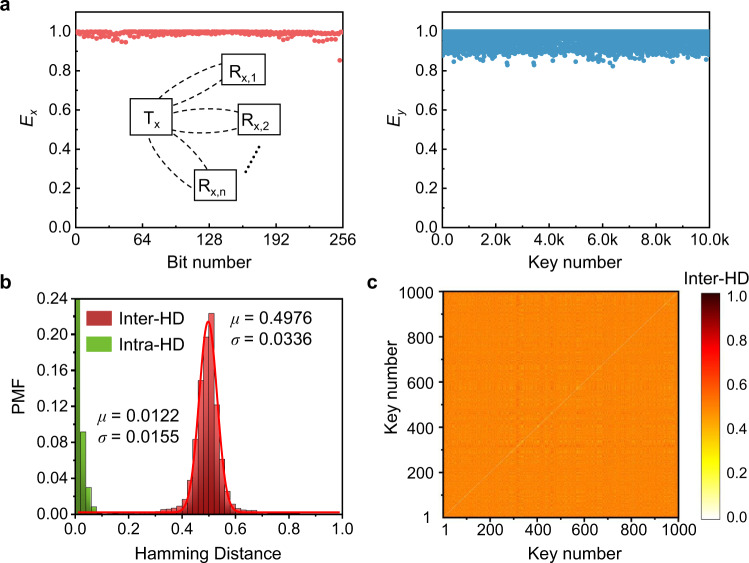
DEP PUF-based authentication. **a** Entropy (*E*_*x*_, *E*_*y*_) analysis of the 256-bits PUF response from 10^4^ stochastically generated PUF instances fro the DEP-based RF PUF system used for the authentication application. We find that entropies in both directions are close to the ideal value of unity (i.e., perfect entropy source). **b** Probability mass function (PMF) of inter-HDs from 10^4^ PUF instances and intra-HDs histograms obtained from 10^3^ PUF instances. The inter-HD is measured at 15 °C, while the intra-HD is measured from −20 °C to 80 °C. A Gaussian fit of the inter-HD histograms is centered at *μ *= 0.4976 with a standard deviation *σ *= 0.0336, which demonstrates excellent uniqueness. This intra-HDs with *σ *= 0.0155 are distributed near the origin, which demonstrates a great reliability. **c** Pairwise evaluation of 10^3^ PUF devices where almost all off-diagonal HDs are close to 0.5.

Next, we also study the scalability of the DEP-based PUF by simulating 10^4^ stochastically generated PUF instances; here the values of *R*, *L*, and *C* were assumed to follow a Gaussian statistical distribution, with tolerances and temperature coefficients based on realistic device database (see Methods for their values and ranges). Figure 6a, b plots entropies and inter-HDs for 10^4^ PUF instances. Surprisingly, even when the number of PUF instances increases by two orders of magnitude, both *E*_*x*_ and *E*_*y*_ are still close to unity and the inter-HDs is excellently fitted by a Gaussian distribution with *μ* = 0.4976 and *σ* = 0.0336; due to the large sample size, the probability mass function (PMF) is used to gauge HD distributions. Figure 6b also reports the reliability analysis (green histograms), showing that the mean value and standard deviation of intra-HDs are close to zero (*μ* = 0.0122 with *σ* = 0.0155). Our simulation results show that important performance metrics including randomness, uniqueness, and reliability remain promising even with a considerable number of PUF instances, thereby demonstrating the excellent scalability of the DEP-based RF PUF. It is also worthwhile noting that the device uniqueness is also regarded as the degree of correlation between the one-dimensional digitized keys of two different PUFs. To clearly illustrate the lack of correlation between arbitrary two PUF units, we plot a pairwise map of 1000 CRPs, where the diagonal line indicates the intra-HD for the PUF instance itself and the off-diagonal points represent the inter-HD values compared to other PUF instances. The sharp contrast of the colormap in Fig. 6c shows a distinct difference between the intra-HD of a specific PUF instance (i.e., ~0) and the inter-HD between two different PUF instances (i.e., small fluctuation around its average of 0.5). In summary, the above discussions verify the possibility of building a lightweight, robust, and scalable solution to secure wireless authorization and access control. We also evaluate the encoding capacity defined as the potential number of codes that can be generated by a PUF instance. The encoding capacity is given by *c*^*n*^. Here, *c* = 2 (i.e., “0” and “1” in a binary digit), and *n* is the key size which is given by *n* = *μ*(1 − *μ*)/*σ*^2^ where *μ* is the mean probability and *σ* is the standard deviation^48^. Based on the results of Fig. 6b, we find *n* = 0.4976 × (1 − 0.4976)/0.0336^2^ ≈ 221 and *c*^*n*^ = 2^221^ = 3.4 × 10^66^. Therefore, the proposed DEP PUF can provide good encryption quality as a hardware security primitive.

**Fig. 7:**
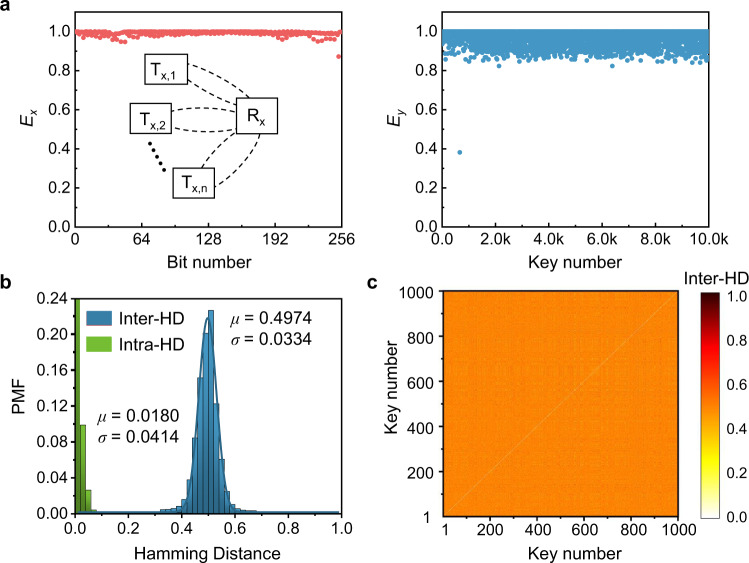
DEP PUF-based near-field communication. **a** Similar to Fig. 6a, but for the application of secure near-field communication (NFC). **b** Probability mass function (PMF) of inter-HD (blue) and intra-HD (green) of the DEP-based RF PUF for the secure NFC applications. The Gaussian fit of the inter-HD histogram is centered at *μ* = 0.4974 with *σ* = 0.0334, manifesting excellent uniqueness. The mean intra-HD *μ* = 0.0180 and the corresponding standard deviation *σ* = 0.0414 show good robustness. **c** Pairwise evaluation of 10^3^ PUF devices shows a perfect uncorrelation level between two arbitrary PUF devices.

In addition to its application in wireless identification, the PUF device proposed here can also be exploited to secure the NFC or low-power wireless sensors, as illustrated in Fig. 1a. When an NFC reader is paired with tags or sensors through inductive coupling, a pulse signal sent from the reader circuit can generate a unique transient response on each receiving device’s capacitor, which can be used as the PUF-based cryptographic key. The PUF-based key detected by the receiving device must be verified before sending out the information stored in its digital memory, thus preventing the participation of the third entity and avoiding the leakage of confidential information. To demonstrate the utility of the proposed device in such an application, we evaluated its merits using a similar numerical experiment to that associated with Fig. 7. We simulated 10^4^ readers with discrepant “−RLC” circuits to interrogate the same receiving device. Here, the lumped elements of the 10^4^ readers are assumed to follow the same Gaussian distribution used before. The entropy plots of Fig. 7a clearly indicate a nearly ideal scenario that guarantees high-quality randomness. The inter-HD shown in Fig. 7b is centered at *μ* = 0.4974 and *σ* = 0.0334, which represent excellent uniqueness and security properties which can also be validated by the pairwise evaluation in Fig. 7c. In addition, the robustness analysis shown in Fig. 7c, quantified by the intra-HD at different temperatures, is also promising with *μ* = 0.0180 with *σ* = 0.0414. Finally, we should note that although this work studies only the case of weak PUFs, the proposed system has the potential to produce strong PUFs. As an example, the RF signature of the output response is sensitive to the challenge’s pulse temporal shape, which enables a set of CRPs to be generated on the same PUF instance, as discussed briefly in Supplementary Note 5.

## Discussion

We have proposed to utilize the extreme sensitivity of PT-symmetric electronic systems near EPs for building a new type of a physical encryption scheme and have shown that these lightweight PUF-based cryptosystems may enable secure authentication and message exchange among the devices. In particular, we have theoretically and experimentally demonstrated that the unprecedentedly large eigenvalues bifurcation observed near divergent exceptional points in higher-order (i.e., third-order and beyond) PT electronic circuits^50,54^ can enhance the randomness, uniqueness, encoding capacity of PUFs generated by inevitable physical differences between devices due to uncontrolled variations in the values of their electronic components. Our results also indicate that this new PUF paradigm can serve as a perfect entropy source or true random number generator for encryption and authentication in enormous wireless communication and identification applications. We expect our result to open a totally new research direction exploiting the implications of non-Hermitian physics, particularly in electronics platforms, for a new class of applications in hardware security.

## Methods

### Experimental measurements

To date, the majority of experimental realizations of PT-symmetry in the RF region rely on the leveraging of VNA since its port impedance can be regarded as perfect negative impedance which may facilitate the system to satisfy the PT-symmetry. However, since the transient responses of the proposed PUF system is the thing of interest in our experiments, a pulse generator is mandatory to replace the VNA to provide the system RF excitations. Thereafter, the perfect negative impedance will be removed. In line with this scenario, a realistic negative impedance with outstanding performance must be implemented to support the PT-symmetry as well as the occurrence of DEP. Here, we design and fabricate a negative impedance converter (NIC) with outstanding performances which can well sustain the realization of EP and DEP. The NIC comprises a unity-gain stable, high-precision and high frequency operational amplifier (OPAMP; OPA817, Texas Instruments Inc.) integrated with proper lumped elements which may exhibit flat negative impedance with relatively small parasitic capacitance within 100 MHz. A photograph of the front and back side of the tank (reader) that consists of the NIC and the corresponding capacitor and inductor (coil) is shown in Supplementary Note 3. From the measured negative impedance of the NIC, the effective negative resistance is ~100 Ω with a relatively small parasitic capacitance of ~25 pF within 100 MHz. Such results demonstrate that the designed NIC can have sufficient quality to construct the PT-symmetric system.

The inductors of different oscillation tanks are realized by coils with fixed self-inductances (*L* = 430 nH for measuring the eigenfrequencies and PUF key extractions of non-EP and DEP architectures while *L* = 580 nH for the EP measurements). When measuring the eigenfrequencies and the PUF key extractions of the traditional wireless systems, the resistance of the “RLC” tanks are fixed at 50 Ω and the quality-factor *γ* is altered from ~1 to ~3 by tailoring the capacitance of the “RLC” tanks from 20 pF to 150 pF. For the measurements of eigenfrequencies of EP and DEP devices, the resistance of the “RLC” tanks are fixed at 33.3 Ω to satisfy the PT symmetry since the total negative impedance of the “−RLC” tank is the product of parallel connection of −100 Ω and −50 Ω (VNA port impedance). To this end, the positive resistance will be replaced with 100 Ω when measuring the PUF keys since without the VNA, the negative resistance is back to −100 Ω. The capacitances for measuring the EP devices’ eigenfrequencies are varied between 470 pF and 47 pF (*γ* between ~1 and ~3) while they are tuned between 1000 pF and 47 pF (*γ* between ~0.6 and ~3) for the measurements of DEP devices. The coupling coefficients for measurements of all three types of setups are fixed at *κ* = 0.65 = 0.92*κ*_DEP_ to make fair comparisons.

## Supplementary information

Supplementary Information

## Data Availability

The data that support the findings of this study are available from the corresponding authors upon request.
