# Supplementary material for: Spectral sensitivity near exceptional points as a resource for hardware encryption
Source: Nat Commun. 2023 Feb 28;14:1145. doi: 10.1038/s41467-023-36508-x (PMC9974995; doi:10.1038/s41467-023-36508-x)
Supplement: Supplementary file 1 — Supplementary Information [file 41467_2023_36508_MOESM1_ESM.pdf]

Spectral sensitivity near exceptional points as a resource for hardware encryption

Supplementary Information

Supplementary Note 1: Time domain characteristics and PUF keys extraction of EP and DEP devices.

To investigate the transient responses of the EP and DEP devices, we first use a function generator to launch pulse signals to the systems. The transient response of the voltage drop across the capacitor of the reader will be probed by the oscilloscope within the time window of $5T_0$ for experiments and $2T_0$ for simulations where $T_0 = 1/f_0 = 2\pi\sqrt{LC}$. Figures S1(a) and (d) demonstrate the transient responses measured from the DEP and EP devices, respectively, and their fast Fourier transforms are shown in Figs. S1(b) and (e). It can be clearly seen that the FFT may straightforwardly depict the three or two resonant peaks of the DEP and EP devices as expected, validating the formation of the third-order and standard PT-symmetric systems. Finally, by repeating the process mentioned above using 16 different RLC tanks, we can have 16 transient responses from the EP and DEP devices ready for the PUF key extractions.

As we discussed, chip resistors, inductors, and capacitors have fabrication tolerances with uncertainty in their values. For example, the surface-mounted chip resistors are fabricated by depositing a resistive film on the ceramic substrate. Since thicknesses of resistive layers may differ during the deposition process (e.g., unintentional impurities, lithographic patterning error, fluctuations in precursor flows and substrate temperature, etc.), the fabrication tolerance typically varies from 0.1% to 5%. Later in our simulations, we make use of the realistic fabrication tolerances and temperature coefficients of the lumped elements that are used in the

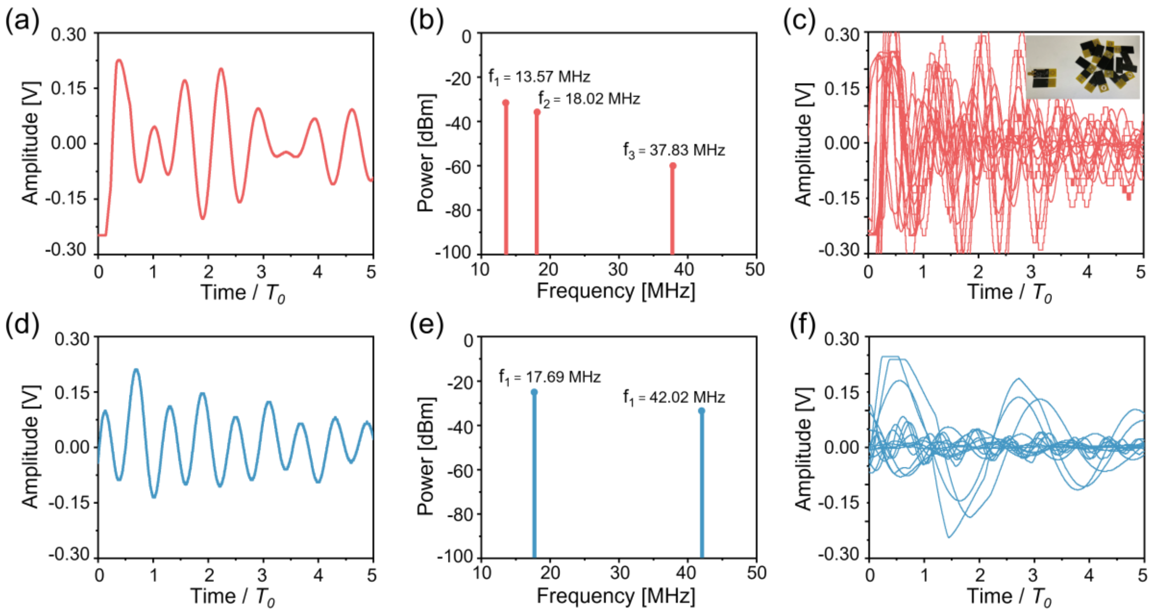

FIG. S1. (a) The output responses of DEP-based circuit measured across the capacitor of the reader. (b) The Fourier transform of (a) in the frequency domain. (c) The temporal responses of 16 DEP-based circuits. The fabricated 16 tags are pictured inside. (d)–(f) are similar to (a)–(c), but for the EP-based circuits.

experiments, which are respectively 5%, and ± 200 ppm / °C. The RF pulse generator launches a pulse RF signal as the challenge to different tags (instances) and the responses are temporal voltage signals measured on the capacitor of the active $-RLC$ oscillator. For example, a constant electromotive force (EMF) at the initial moment of $\boldsymbol{\Psi}(\tau=0)=(1,0,0,0,0,0)^T$, the evolution of charges stored on capacitors of sixteen randomly selected PUF instances from measurements is presented in Figs. S1(c) and (f). It can be seen that the temporal responses are unique and non-repeatable. Such uniqueness found in the analog form can be reserved even after mapping to the digital cryptographic keys [see Fig. 1(a) in the main text].

The extraction of digital cryptographic keys is detailed below. First, the temporal response of voltage across the capacitor of the $-RLC$ oscillator (i.e., reader for wireless identification) is recorded in the first five periods for the simulations, as shown in Fig. S1(c)

(The transient responses obtained from simulation are within two periods). For secure communication applications, the temporal response is recorded on the capacitor of the receiver. Then, the temporal response is properly scaled and normalized in the range between 0 and 1 and is uniformly discretized into 64 data points (R_1 – R_{64}) in the time domain, as sketched in Fig. 1(b) in the main text. Each data point R_i is digitized into 4-bit binary codes ranging from 0000 to 1111, depending on its floating number value. A 4-bit binary code 0000 will be assigned if the normalized data point is smaller than 0.0625. Subsequently, a 256-bit CRP sequence can be generated based on the temporal response, originally stored in an analog form. Here to have a statistical analysis of the bitmap, we choose 100 from 10000 simulated PUF keys as a sufficient sample size to repeat the same procedures; a CRP map (i.e., bitmap) can be obtained, as shown in the inset of Fig. 1(a) in the main text and Figs. S2 and S3. We note that the encryption quality provided by the proposed PUF can be further improved by expanding the size of the bitmap. For example, the discretization process can be ameliorated by choosing a high sampling rate to gain more individual data points. In addition, extending the binary-coded length to 8 bits or 16 bits can also increase the size of the bitmap.

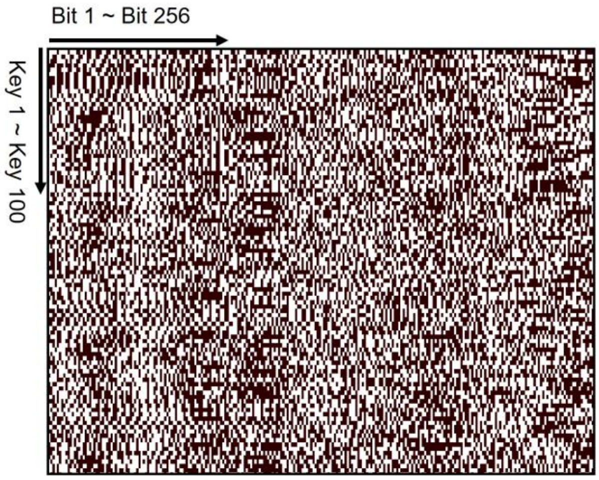

FIG. S2. Bitmap obtained from 100 PUF keys for the wireless authentication/identification application.

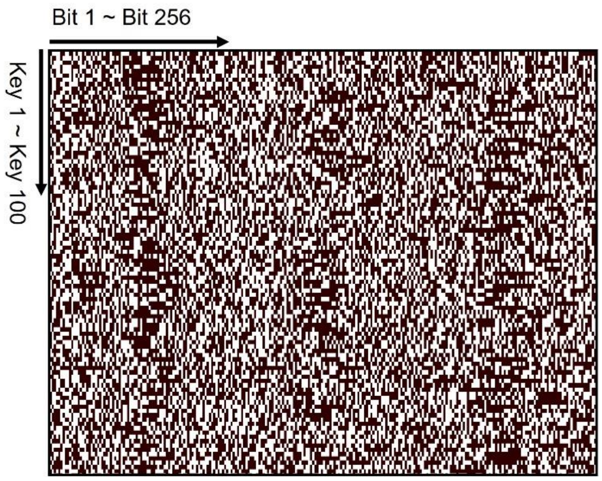

FIG. S3. Bitmap obtained from 100 PUF keys the secure communication application.

Supplementary Note 2: Eigenfrequencies of third-order PT-symmetry

Considering the circuit diagram shown in the left panel of Fig. 2(a) in the main text, which is formed by active ($-RLC$), neutral (LC), and passive (RLC) oscillators. One can apply Kirchhoff's laws to such an electronic circuit and, after some manipulation, derive the Liouvillian eigenvalue problem, given by [1]:

$$\mathcal{L}\mathbf{\Psi} = \frac{d\mathbf{\Psi}}{d\tau},$$
$$\mathcal{L}_{\text{3rd PT}} = \begin{pmatrix} 0 & 0 & 0 & 1 & 0 & 0 \\ 0 & 0 & 0 & 0 & 1 & 0 \\ 0 & 0 & 0 & 0 & 0 & 1 \\ -\frac{1-\kappa^2}{1-2\kappa^2} & \frac{\kappa}{1-2\kappa^2} & -\frac{\kappa^2}{1-2\kappa^2} & \frac{1}{\gamma}\frac{1-\kappa^2}{1-2\kappa^2} & 0 & -\frac{1}{\gamma}\frac{\kappa^2}{1-2\kappa^2} \\ \frac{\kappa}{1-2\kappa^2} & -\frac{1}{1-2\kappa^2} & \frac{\kappa}{1-2\kappa^2} & -\frac{1}{\gamma}\frac{\kappa}{1-2\kappa^2} & 0 & \frac{1}{\gamma}\frac{\kappa}{1-2\kappa^2} \\ -\frac{\kappa^2}{1-2\kappa^2} & \frac{\kappa}{1-2\kappa^2} & -\frac{1-\kappa^2}{1-2\kappa^2} & \frac{1}{\gamma}\frac{\kappa^2}{1-2\kappa^2} & 0 & -\frac{1}{\gamma}\frac{1-\kappa^2}{1-2\kappa^2} \end{pmatrix}, \tag{S1}$$

where $\mathbf{\Psi} \equiv \left(q_1, q_2, q_3, \dot{q}_1, \dot{q}_2, \dot{q}_3\right)^T$, the dimensionless gain-loss parameter $\gamma = R^{-1}\sqrt{L/C}$ referring to the non-Hermiticity of the PT system, the normalized coupling strength $\kappa = M/L$,

and M is the mutual inductance of two coil antennas with self-inductance of L. In addition, $\tau = \omega_0 t$, and $\omega_0 = 1/\sqrt{LC}$. Throughout this study, the time-harmonic notation $\exp(-i\omega\tau)$ is adopted. When doing the circuit analysis, we assume the RF pulse generator is removed. Therefore, the effective Hamiltonian of the system can be written as $H_{\text{eff}}\mathbf{\Psi} = i\mathcal{L}\mathbf{\Psi} = i\dfrac{d\mathbf{\Psi}}{d\tau}$, which is non-Hermitian ($H_{\text{eff}}^{\dagger} \neq H_{\text{eff}}$) and symmetric with respect to the $\mathcal{PT}$, namely $\left[\mathcal{PT}, H_{\text{eff}}\right] = 0$, with

$$\mathcal{P} = \begin{pmatrix} \mathbf{J} & 0 \\ 0 & \mathbf{J} \end{pmatrix} \text{ and } \mathcal{T} = \begin{pmatrix} \mathbf{I} & 0 \\ 0 & -\mathbf{I} \end{pmatrix}\mathcal{K}, \tag{S2}$$

where $\mathbf{J}$ is the 3×3 anti-diagonal identity matrix, $\mathbf{I}$ is the 3×3 identity matrix, and $\mathcal{K}$ conducts the operation of complex conjugation. These operations in conjunction leave the system unaltered. The system has six eigenvalues or eigenfrequencies, which can be derived from the secular equation as (in units of ω_0):

$$\begin{aligned} &\omega_{1,2} = \pm 1, \\ &\omega_{3,4} = \pm\sqrt{\frac{2\gamma^2 - 1 - \sqrt{1 - 4\gamma^2 + 8\gamma^4\kappa^2}}{2\gamma^2\left(1 - 2\kappa^2\right)}}, \quad \text{and} \quad \omega_{5,6} = \pm\sqrt{\frac{2\gamma^2 - 1 + \sqrt{1 - 4\gamma^2 + 8\gamma^4\kappa^2}}{2\gamma^2\left(1 - 2\kappa^2\right)}}. \end{aligned} \tag{S3}$$

By inspecting Eq. (S3), one can identify three different regimes, separated by the exceptional points $\gamma_{\text{EP},\pm} = \sqrt{1 \pm \sqrt{1 - 2\kappa^2}}\,/\,(2\kappa)$. The dynamics (time-transient response) of the third-order PT system is a linear combination of all eigenmodes $\mathbf{\Psi}(\tau) = \sum_{n=1}^{6} c_n \Phi_n e^{-i\omega_n\tau}$, where the coefficient c_n depends on the initial condition, and eigenfrequencies and eigenmodes can be expressed as follows:

$$\Phi_n = \left(X_n, Y_n, Z_n, -i\omega_n X_n, -i\omega_n Y_n, -i\omega_n Z_n\right)^T, \tag{S4}$$

where

$$X_n = 1,$$
$$Y_n = \frac{i\omega_n + \gamma(1-\omega_n^2)}{\gamma\kappa\omega_n^2}, \tag{S5}$$
$$Z_n = -\frac{\omega_n[(\kappa^2-1)\omega_n^2+1] + i\gamma[(2\kappa^2-1)\omega_n^4-(\kappa^2-2)\omega_n^2-1]}{\kappa^2\omega_n^2(\omega_n+i\gamma)}.$$

We note that in the exact PT-symmetric phase, one may observe an oscillatory motion consisting of the superposition of three harmonics [Fig. S1]. In the broken phase, due to the positive imaginary part of complex eigenfrequencies, the eigenmodes grow exponentially in time, and, thus, the system exhibits unstable, underdamped behavior. Beyond the point of critical damping, the eigenfrequencies are purely imaginary, and the eigenmodes are either exponentially growing or decaying in the temporal responses. Such a phase is an overdamped mode, with exponential responses arising in the temporal dynamics of charges and displacement currents.

Figure S4 reports distributions of eigenfrequencies of the standard PT-symmetric electronic system and the traditional telemetry system using a coil antenna under the same Gaussian distributions in impedance values of lumped elements in the *RLC* oscillator. The result manifests that the PUF properties of the traditional or EP-based telemetry systems exhibit much-reduced randomness when compared with the DEP-enabled counterpart in Fig. 2(a) in the main text.

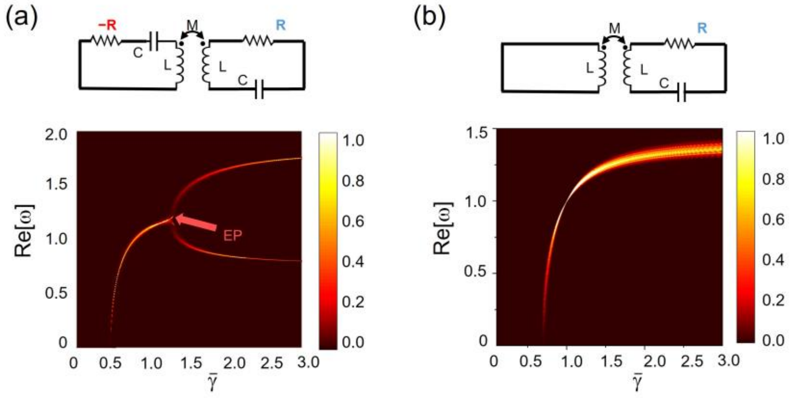

FIG. S4. Similar to Fig. 3(a) in the main text, but for (a) the standard PT-symmetric electronic system and (b) the traditional telemetry system.

Supplementary Note 3: Design of Negative Impedance Converter

In our experimental validation, a negative impedance converter (NIC) with outstanding performances that can sustain the realization of EP and DEP was designed and fabricated. The circuit structure is shown in the inset of Fig. S5(b), which comprises a unity-gain stable, high-precision, and high-frequency operational amplifier (OPAMP; OPA817, Texas Instruments Inc.) integrated with proper lumped elements. Figure S5(a) shows the photograph of the front and back sides of the $-RLC$ tank (reader) that consists of the NIC and the corresponding capacitor and inductor (coil). Figure S5(b) plots the measured negative impedance of the NIC, from which we could see the effective negative resistance is $\sim 100\ \Omega$ with a relatively small parasitic capacitance of within 100 MHz.

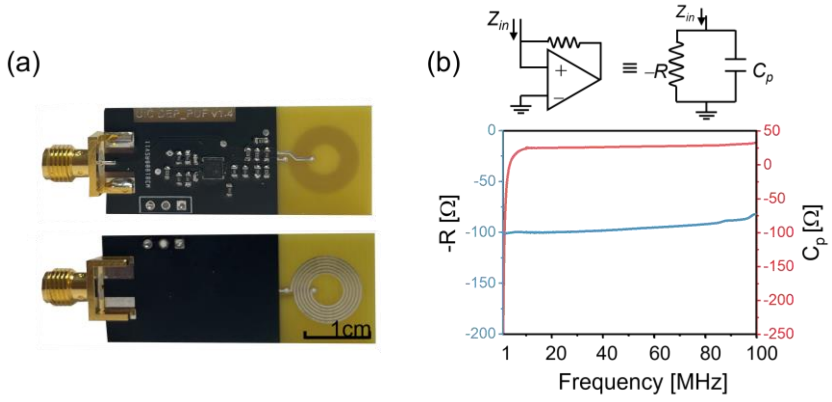

FIG. S5. (a) Pictures of the tank used for the DEP and EP PUF measurements. (b) The extracted effective negative resistance and parasitic capacitance from the measured negative impedance of the NIC.

Supplementary Note 4: National Institute of Standards and Technology (NIST) randomness tests

An excellent PUF can be regarded as a true random number generator (TRNG) capable of generating perfectly random number sequences. The randomness of bit sequences is of great importance in cryptographic applications. The NIST randomness tests are developed to determine whether a binary sequence generated by a source is genuinely random. The tests include 15 contents in total, which use probability functions (e.g., complete and incomplete gamma functions, error functions, etc.) to examine the binary sequences in different aspects. For example, the frequency test, also referred to as the monobit test, mainly focuses on the proportion of zeros and ones of the bitstring. It is easy for a sequence to pass this test since it only requires that distributions of zeros and ones are the same. However, the same sequence may not be random if its first half bits are all zeros or ones or if the occurrences of zeros and ones are periodical. To avoid this scenario, other NIST tests are required to be passed. For these tests, P-value is the probability that a perfect random number generator would have produced a sequence less random than the test sequence, given the kind of nonrandomness assessed by

Table I. P-values of PUF keys obtained from DEP, EP, and non-EP devices.

NIST	Sim. Identification		Sim. Secure Communication		Exp. DEP Identification		Exp. EP Identification		Exp. Non-EP Identification	
	P-values	Pass?	P-values	Pass?	P-values	Pass?	P-values	Pass?	P-values	Pass?
Frequency	0.7929	Yes	0.9601	Yes	0.8513	Yes	1.0000	Yes	0.1336	Yes
FB	0.3697	Yes	0.3526	Yes	0.3408	Yes	0.2640	Yes	0	No
Runs	0.1974	Yes	0.5253	Yes	0.3596	Yes	0.3913	Yes	0.0001	No
LOR	0.1396	Yes	0.0815	Yes	0.0725	Yes	0.0168	Yes	0.0014	No
FFT	0.9510	Yes	0.9813	Yes	1.0000	Yes	1.0000	Yes	1.0000	Yes
NOT (m=5)	0.9685	Yes	0.9675	Yes	0.9863	Yes	0.9967	Yes	0.9962	Yes
Serial (m=4)	0.5815	Yes	0.5047	Yes	0.3006	Yes	0.0514	Yes	0	No
AppEn (m=3)	0.5506	Yes	0.4518	Yes	0.2028	Yes	0	No	0	No
Cum. Sum.	0.2984	Yes	0.5880	Yes	0.2974	Yes	0.3859	Yes	0	No

the test [2]. Typically, the PUF can be regarded as a true random generator if all tests' P-values are greater than 0.01. Table I reports the results of NIST tests of the PUF keys generated from simulations when the DEP PUF is used for both identification and secure communication applications and the experimental measurements when DEP, EP, and traditional wireless setups are used for identification purposes. Here we randomly choose 100 PUF keys from the simulation to perform the tests. From the table, we may obtain that the DEP can be indeed regarded as a true random number generator with higher P-values passing the tests.

Supplementary Note 5: The influence of pulse-to-pulse variations and time-modulated pulse signals on DEP PUF

The small pulse-to-pulse variations do indeed exist during the measurements, which, however, will not significantly degrade the performances of the proposed DEP PUF. Although the intra-HDs in our paper tend to investigate the temperature stability of the DEP PUF, at the same time, the pulse signals as input challenges to the DEP PUF instances will also have slight fluctuations for these intra-HD measurements, which should also be considered as different operating conditions. Therefore, the experimentally measured intra-HDs are the products of the combined effects of different temperatures and different pulse signals of the DEP PUF, by which the results have rendered a high consistency among different operating conditions. Thus, tiny pulse-to-pulse variations will not significantly influence the DEP PUF.

In addition, we also study the unique transient responses that the DEP PUF may provide concerning the different shapes of pulse signals. As illustrated in Fig. S6, the transient responses will be altered entirely once the pulse or step signal has been added with specific time modulations. By doing so, we may obtain a large number of CRPs by a single PUF instance, and therefore, the DEP PUF may be regarded as a strong PUF.

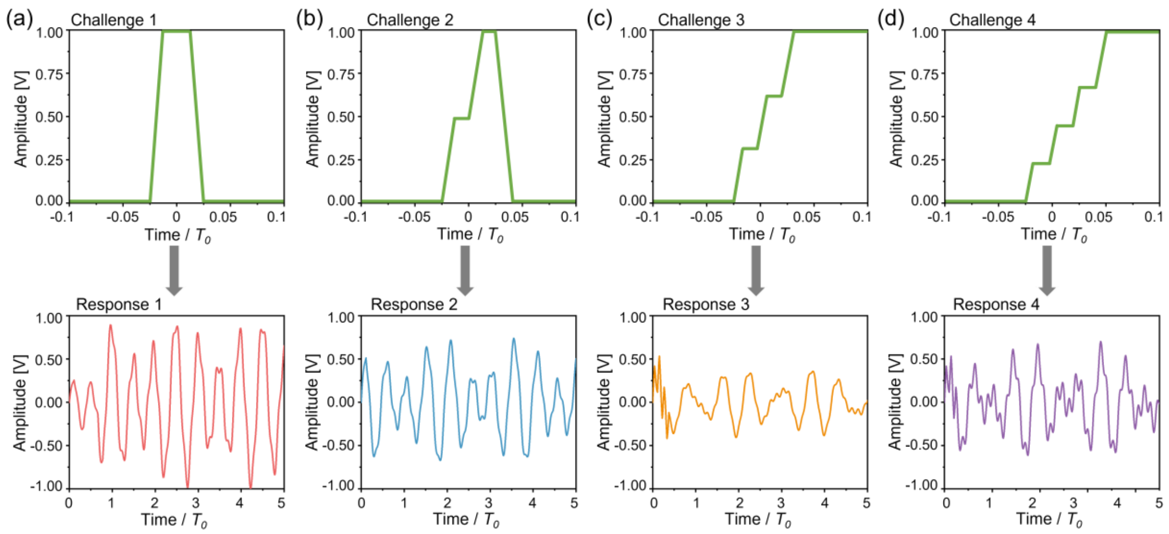

FIG. S6. (a) The challenge-response pair of the DEP PUF with a normal pulse signal was applied. (b), (c) and (d) are the challenge-response pairs of the same DEP PUF instance when the RF "challenge" has specific time modulations.

Supplementary References

[1] J. Schindler, A. Li, M. C. Zheng, F. M. Ellis, and T. Kottos, Experimental Study of Active LRC Circuits with PT Symmetries, *Phys. Rev. A* **84**, 4 (2011).

[2] Rukhin, A. et al. A Statistical Test Suite for Random and Pseudorandom Number Generators for Cryptographic Applications. NIST Special Publication 800-822 (National Institute of Standards and Technology, 2010)
